# Ayurveda: Between Religion, Spirituality, and Medicine

**DOI:** 10.1155/2013/952432

**Published:** 2013-11-28

**Authors:** C. Kessler, M. Wischnewsky, A. Michalsen, C. Eisenmann, J. Melzer

**Affiliations:** ^1^Department of Internal and Complementary Medicine, Immanuel Hospital and Institute of Social Medicine, Epidemiology & Health Economics, Charité-University Medical Center, Research Coordination, Königstraße 63, 14109 Berlin, Germany; ^2^eScience Center, University of Bremen, Universitätsallee, 28359 Bremen, Germany; ^3^Graduate School in History and Sociology, Bielefeld University, 33615 Bielefeld, Germany; ^4^Institute of Complementary Medicine, University Hospital Zurich, 8001 Zurich, Switzerland; ^5^Department for Psychiatry, Psychotherapy and Psychosomatics, Königin-Elisabeth-Herzberge Hospital, 10365 Berlin, Germany

## Abstract

Ayurveda is playing a growing part in Europe. Questions regarding the role of religion and spirituality within Ayurveda are discussed widely. Yet, there is little data on the influence of religious and spiritual aspects on its European diffusion. *Methods*. A survey was conducted with a new questionnaire. It was analysed by calculating frequency variables and testing differences in distributions with the **χ** ^2^-Test. Principal Component Analyses with Varimax Rotation were performed. *Results*. 140 questionnaires were analysed. Researchers found that individual religious and spiritual backgrounds influence attitudes and expectations towards Ayurveda. Statistical relationships were found between religious/spiritual backgrounds and decisions to offer/access Ayurveda. Accessing Ayurveda did not exclude the simultaneous use of modern medicine and CAM. From the majority's perspective Ayurveda is simultaneously a science, medicine, and a spiritual approach. *Conclusion*. Ayurveda seems to be able to satisfy the individual needs of therapists and patients, despite worldview differences. Ayurvedic concepts are based on anthropologic assumptions including different levels of existence in healing approaches. Thereby, Ayurveda can be seen in accordance with the prerequisites for a Whole Medical System. As a result of this, intimate and individual therapist-patient relationships can emerge. Larger surveys involving bigger participant numbers with fully validated questionnaires are warranted to support these results.

## 1. Introduction

Ayurveda, a form of Traditional Indian Medicine (TIM), literally translates from Sanskrit to “knowledge of life” or more precisely “systematic knowledge of the lifespan” [1]. Ayurveda is a Whole System of Medicine (WMS) [2–5]. In its South Asian countries of origin it has been practiced for more than 2000 years in an unbroken tradition and is thus one of the oldest WMS of mankind [6]. Ayurveda is fully recognized by the World Health Organization (WHO) as a medical science analogous to Traditional Chinese Medicine (TCM) and has amassed an enormous wealth of empirical healing knowledge. (Proto)scientific concepts have had a firm place in mainstream Ayurvedic medicine ever since around the beginning of the common era with the emergence of the “classic texts” (e.g., Caraka Samhita [7, 8]) and are centered around designated disciplines of logic and methodology [9]. In India and some neighboring countries, Ayurvedic medicine is officially and legally recognized as on par with conventional medicine. It is used in an area with more than 1.4 billion people as a broad system of medicine [10, 11]. The importance of Ayurveda in modern South Asian health care setups is reflected by the following figures: in India alone above 400,000 registered Ayurvedic physicians practice Ayurveda [12] and there are more than 250 universities and colleges where Ayurvedic medicine is systematically taught as a 4–6-year university degree program [13]. In its diagnostic and therapeutic approaches Ayurveda is steeped in the principles of salutogenesis [14] Primary, secondary, and tertiary prevention, patient self-empowerment, and self-efficacy play crucial roles in the holistic and multidimensional Ayurvedic approach to healing [15]. Ayurveda not only is a WMS but also incorporates eclectic philosophies of life that have helped to shape complex theories about health and disease over more than three millennia, including philosophical, epistemological, and spiritual dimensions. For example, Ayurveda postulates a paradigmatic harmony of physiological, psychological, social, and environmental factors of the human microcosm and the universal macrocosm [16, 17].

In addition to its key role in Asian health care systems, it is playing a growing role in Complementary and Alternative Medicine (CAM), especially in integrative settings in Europe and North America. For instance, in Germany, Austria, and Switzerland Ayurveda is one of the fastest growing CAM methods [18]. An internet search for “Ayurveda” yields >7,400,000 entries in Google [19]. In 2011 the establishment of the German Medical Doctors Association of Ayurvedic Medicine (DÄGAM) took place [20]. In several training institutions throughout Germany professional development and training opportunities certified by various state-level German Medical Doctors' Associations are being offered (e.g., in Bavaria, Berlin, North Rhine-Westphalia, Schleswig-Holstein, Hessen, Hamburg, and Rhineland-Palatinate). Yet there is no national certificate for Ayurveda. Important areas of discussion surrounding the character of Ayurveda include (a) its underlying core concepts for diagnosis and therapy, (b) ultimate therapeutic aims, and (c) demarcation from other South Asian traditional medical systems (e.g., Siddha, Unani-Tibb) and modern western medicine and remain largely unanswered [17]. Inquiries regarding the importance of religion and spirituality within medical contexts have been posed repeatedly in Indology, Sociology, Anthropology, Religious Studies, and Medical Sciences [18, 21, 22]. Whole Medical Systems (WMS) are by definition complete and coherent systems of medical theory and practice that have evolved and continue evolving, in different regions, cultures, and time periods around the globe. They have evolved relatively independent of modern western medicine, for example, Traditional European Medicine (anthroposophy, homeopathy, and naturopathy), Traditional Chinese Medicine (TCM), Tibetan Medicine, or Arabian systems of medicine [23–29].

Concerning Ayurveda, two main opposing positions can be observed: [16] (a) supporters of “scientific” Ayurveda state that it has always been an empirical medical system in which religious and spiritual speculations are mere interpolations, alien to the system, or (b) supporters of “traditional” Ayurveda state that religious and spiritual elements have always been integral components of Ayurveda as a WMS. These positions are, however, not mutually exclusive.

There is growing acceptance and demand for Ayurveda in western countries and there are currently more than 2500 online publications on Ayurvedic therapies in PubMed [30] and greater than 52,000 referenced Ayurveda research articles in the Indian digital database DHARA (Digital Helpline for Ayurveda Research Articles) [31]. It is hypothesized that spirituality might be a main attractor for the increasing popularity of Ayurveda [32]; however, there is still little scientific evidence regarding the influence of religious and spiritual elements on the diffusion and implementation of modern hybrid forms of Ayurveda [33–35].

This is striking because spirituality has already entered discussions in neurobiology [36] and most of all quality of life (QoL) research [37], especially in chronic diseases [38–44]. However, cultural and spiritual attractors of nonwestern CAM have been discussed in recent years [45, 46] and are beginning to be researched [47, 48]. The rather late awareness of spiritual aspects in CAM might be due to the impact that the methodology of Evidence-based Medicine (EbM) had on the medical system as such and in particular on research initiatives in CAM. More recently, after CAM research has managed to close some evidence gaps, researchers have become aware of the necessity to conduct research focused not only on specific evidence but also on unspecific or contextual or patient-centred aspects (related to CAM) [49–52]. This is by no means in opposition to EbM because one of its founders defined EbM as the integration of (a) the best research evidence with (b) clinical expertise and (c) patient values [53]. However, clinical research had focused predominantly on the two former aspects until recently.

In order to explore the general role of religion and spirituality specifically within the field of Ayurveda, a new questionnaire was developed. While existing questionnaires, for example, the Spiritual Perspective Scale [54, 55], the S-PRIT [56], the FACIT-Sp [57], the Spiritual Well-Being Scale [58], Aspects of Spirituality [59], the SpREUK [60], the Health and Religious Congruency Scale [61] and others [62–68] would be useful for further analysis, the objective of this pilot survey was to focus on the specificities of the complex field of Ayurveda in a western setting, leaving the definition of spirituality as open as possible. Spirituality and religion were thereby not used as analytical but as emic (ethno)categories [69–71]. This questionnaire was distributed among patients accessing and therapists offering Ayurveda in German-speaking countries.

### 1.1. Hypothesis

To shed some light on the influence and meaning of religious and spiritual aspects on the diffusion and implementation of Ayurvedic practices in Europe the following hypotheses were formulated to the survey a priori.

- *Hypothesis 1.* Participants who apply Ayurveda as a therapist or receive Ayurveda as a therapy are religious and/or spiritual. Ayurveda is perceived as a healthcare approach which incorporates religious and spiritual demands.
- *Hypothesis 2.* For patients and therapists, principles of Ayurveda and modern science are not in conflict. Concepts of religion, spirituality, and science can be integrated.
- *Hypothesis 3.* Elements from South Asian cultures, religions, and philosophies are supposed to have an effect on the results of Ayurvedic therapies.
- *Hypothesis 4.* Women are more open to religious and spiritual aspects in the case of Ayurvedic therapists and patients than men.

## 2. Methods

### 2.1. Survey

To test these hypotheses a questionnaire was developed and distributed among patients and therapists in western Ayurvedic health care settings in Frankfurt a. M., Birstein, Passau, Bremen, Hanover, Zurich, and Vienna. These settings included (a) private Ayurvedic practices, (b) the International Ayurveda Symposium in Birstein, and (c) direct contacts of the corresponding author. To rule out any potential selection bias of the participants, questionnaires were given to the first sequential 300 eligible persons contacted.

To be included participants had to be ≥18 years of age. Patient participants had to have had ≥1 experience with Ayurvedic therapies and therapist participants had to have had at least one course of institutionalized Ayurvedic training and had to offer Ayurvedic therapies or have a plan to do so at the time of inclusion (details about the individual training duration were not further assessed).

Patients were excluded if they suffered from a life threatening disease, in order to avoid systematic bias/confounders due to a “last exit mentality” which can influence the overall compliance with respect to their choice of therapies and therapists.

The survey with anonymized questionnaires, part of a master thesis for the corresponding author, was performed at the Institute for Indology and Tibetology, Philosophical Faculty, University of Göttingen, Germany. Of note this is not a clinical study, and according to university procedures therefore no ethical approval was mandatory and informed consent, anonymized questionnaires, and respect of data privacy were sufficient.

### 2.2. Construction of the Questionnaire

Firstly, a preliminary questionnaire considering content validity, internal consistency, criterion validity, construct validity, and reproducibility was developed [72].

The items for the preliminary questionnaire version were derived from three sources: (1) exploratory interviews with expert representatives, (2) as discussed in the research literature, and (3) items inspired by existing questionnaires in the field (e.g., “Aspects of Spirituality,” see above). This preliminary version of the questionnaire was pretested with 10 test persons accustomed to filling out questionnaires, to gain information on reliability and validity aspects. The questionnaire was then modified based on the received feedback and reexamined. It was then modified and approved by expert representatives and scholars from various disciplines (Medicine, Indology, Religious Sciences, Informatics, and Sociology). This resulted in a final version of the questionnaire to be distributed to the target group in its finalized version. Therefore the underlying questionnaire might be regarded as a “standard” questionnaire in the sense of Olsen [73]. A validated questionnaire in the traditional sense was not possible, since we could not compare this instrument against a gold standard, as such a gold standard for Ayurveda as a Whole Medical System this context does not yet exist [58].

The final version of the questionnaire included a section for sociodemographic baseline data and 50 questionnaire items. The majority of the items are scored on a 5-point Likert scale ranging from “total” disagreement to “total” agreement (0–4) or on a 3-point Likert scale (i.e., “yes,” “no,” and “do not know”). In order to obviate the problem of acquiescence bias, we designed a scale with balanced keying (an equal number of positive and negative statements), while possible distortions through central tendency and social desirability are more difficult to control.

### 2.3. Statistics and Validation

All returned questionnaires underwent statistical analysis. For descriptive statistics each item was analyzed separately and in some cases item responses were summed to create a score for a group of items. The frequencies of the various variables were calculated. Differences in frequency distributions were tested with the *χ* ^2^-Test. Principal Component Analyses with Varimax Rotation and Kaiser Normalization were used to represent the main structural features of the multivariate data set by a smaller number of attributes. This is achieved by transforming data from the original coordinate system (i.e., spanned by the original attributes) into a different coordinate system where the variables are linearly independent. The factor loading, a standardized scoring coefficient, was used to determine the contribution of a variable to a particular factor. Variables with rotated absolute factor values >0.5 (or <−0.5) for a particular factor were considered significant contributors for that factor (see Table 3. The 12 variables are replaced by 4 factors: for example, the variables “Ayurveda = Spirituality?,” “Ayurveda = Philosophical system?,” and “Ayurveda = Way of life?” have rotated absolute factor values >0.5 for factor 1, which expresses a strong correlation between these variables; all other variables have factor values ≤0.5 or ≥−0.5 with respect to factor 1; therefore they do not significantly contribute to this factor). Negative rotated absolute factor values express inverse correlations. We used 10 or more test persons per item in connection with multivariate analyses. Ten test persons per 1 item is a well-known rule of thumb for the number of instances (data sets) in connection with knowledge discovery processes, that is, multivariate analyses. Based on reliability analyses inner consistencies and discriminatory power were tested. A significance level of *P* < 0.05 was taken as a basis. Calculations were performed with NCSS (version 2007) and SPSS (version 19).

The validation of questionnaires in general is based on methods of the classical test theory and factor analyses for the design of questionnaire items. Factor analysis was one of the central methods for the evaluation of this questionnaire. It serves for the grouping of parameters and for the partial validation of this questionnaire. The a priori allocation of different subject areas was tested by factor analyses. For each subject area a factor analysis was calculated to find out whether the chosen subject area captures the construct or whether the existence of several factors hints at the existence of different subconstructs. We used the Principal Components Analysis as extraction method. As a support for finding out the number of factors of a subject area (=number of subconstructs) we used the Kaiser-Guttman criterion [74] (number of factors to be extracted = number of the factors with eigenvalue >1) and the scree test of the eigenvalue course [75].

## 3. Results

Overall 300 questionnaires were distributed (120 in private practices, 130 at the International Ayurveda Symposium in Birstein, and 70 through direct contacts of the corresponding author). 140 completed questionnaires were returned, exactly (and coincidently) 70 from patients and 70 from therapists (53 from private practices, 45 from the 7th International Ayurveda Symposium in Birstein, and 42 from professional contacts of the corresponding author). Following the sociodemographic background, the results of the questionnaire will be summarized in order of the respective hypotheses.

Parts of the results are presented as pooled data from patients and therapists wherever there is no significant difference between the two groups.

### 3.1. Baseline Data

*Sociodemographic.* Among the participants of the survey a significant difference in sociodemographic data was only found for profession but not for age, gender, education, income, or location (Table 1).

*Other*. Four survey participants (all patients) had only 1 experience with Ayurveda at the time of the interview. The individual training range from the surveyed therapists ranges widely from below three months to a 4–6-year academic Ayurvedic training in South Asia.

### 3.2. Findings Related to Hypothesis 1

65% of the respondents belong to a religion and describe themselves as religious/spiritual. 81% describe the influence of religion and spirituality on their daily life as important. 73% consider Ayurveda to be a form of spirituality (76% of therapists, 57% of patients), but only 11% think of Ayurveda as a religion (findings not shown).

Traditional Christian values and beliefs are confirmed (e.g., 77% believe in God), but in addition a majority also believe in non-Christian concepts (karma 66%, rebirth 64%, and transmigration of the soul 58%). Patients adhere more to traditional Christian values and beliefs than therapists; for instance, a belief in a Christian god can be observed among 83% of patients and 71% of therapists (*P* = 0.107). Yet at the same time therapists adhere more to traditional South Asian values and beliefs: 84% of therapists and 59% of patients believe in karma (*P* = 0.003), 74% of therapists and 54% of patients believe in rebirth (*P* = 0.009). A general affinity for South Asian religions is noticeable. 71% share a fascination for Buddhism and 38% for Hinduism (no significant differences between patients and therapists). 49% find Christian religions to be lacking mystical elements that can be better served by Buddhism or Hinduism. 43% think that South Asian religions can respond better to prevailing problems than western religions. 60% of all respondents believe that disease is conditioned through karma while 95% are convinced that faith and belief are important prerequisites for healing. Still 81% think that divine power and karma (66%) are important healing factors and 67% have prayed (74% among therapists, 61% among patients (*P* = 0.138)).

Three “groups of believers” can be delineated: (1) a group, whose members simultaneously believe in karma, nirvana, a universal soul, transmigration of the soul, and rebirth; (2) a group with a statistical relation between believing in the god, the devil, and angels; and (3) a group, characterized by simultaneous beliefs in a metaphysical sense of life and god(s).

The most prominent aspects of traditional Christian spirituality and of South Asian spirituality derived from this data are (1) belief in God (Bonferroni adjusted *P* value (adj. *P*) *P* < 0.001), (2) belief in divine beings (adj. *P* < 0.001), and (3) belief in rebirth (adj. *P* = 0.010). Only 3 patients and 1 therapist declared themselves as nonreligious. Nevertheless 3 of these believe in a cosmic soul, karma, rebirth, sense of life, divine beings, or transgression of soul, so only 1 “non-believer” remains in total.

### 3.3. Findings Related to Hypothesis 2

100% of all participants (valid cases) consider Ayurveda to be a health doctrine, 95% to be a medical system, and 93% to be a science. 80% relate it to a philosophical system (87% among therapists, 55% among patients (*P* = 0.035)), while 73% of all respondents consider Ayurveda to be a form of spirituality. However, only 11% consider Ayurveda to be a religion (Table 2). 76% believe that Ayurvedic therapists have functions related to spiritual guidance (therapists 79%, patients 73% (*P* = 0.641)). Though a majority (93%) of respondents consider Ayurveda to be a science, only 28% think that Ayurveda is scientific in a modern western sense. 59% see Ayurveda as a complement to modern medicine, while more than 25% think that it should be used exclusively. Only about 30% state that Ayurveda should be analysed through scientific studies (therapists 29%, patients 32% (*P* = 0.260)). However, 76% think that medical aspects of Ayurveda are more important than religious and/or spiritual aspects (therapists 74%, patients 78% (*P* = 0.635)). 25% consider schooling in modern medicine to be a negative influence on the religious and spiritual characteristics of the Ayurvedic therapist.

The 12 variables in Table 3 could be reduced to 4 different factors: (1) factor 1 comprises the variables designating Ayurveda to have a spiritual nature, to be a philosophical system, and to be a way of life; (2) factor 2 accounts for the correlation that it is a religion, a religious doctrine, and esoteric; (3) factor 3 sees it as a medical system, a science, and a philosophy of life; and (4) factor 4 pulls together the perceptions of Ayurveda as a complement to modern medicine and as scientific in a modern western sense (Table 3).

### 3.4. Findings Related to Hypothesis 3

65% of respondents believe that Ayurveda can be expediently practiced in the West, detached from South Asian culture, religion, and philosophy. At the same time 66% believe that Ayurvedic experts from South Asia should participate in teaching the medical system (which actually occurred in 87% of the cases). Almost 50% of the participants are convinced that Ayurvedic schooling should include at least one study visit to South Asia. 71% have the opinion that Ayurveda therapists should educate their patients in fundamental concepts of Ayurveda during the therapy. 50% of the interviewees think that basic knowledge about South Asian culture is important for patients. 61% agree with the statement that Ayurvedic therapists should sympathize with South Asian culture, religion, and philosophy, while 67% feel attached to South Asian culture, religion, and philosophy (80% among therapists, 55% among patients (*P* = 0.003)). 70% of the participants (therapists 73%, patients 67% (*P* = 0.476)) think that following an Ayurvedic lifestyle attitude is important, while 57% actually practice such a lifestyle (therapists 69%, patients 46% (*P* = 0.016)). A majority of the respondents feel well acquainted with the concepts of reincarnation, karma, migration of the soul, nirvana, attachment, atman, brahman, enlightenment, and Buddhism. 30% of the interviewees think that exact knowledge of the precise meaning of certain Ayurvedic Sanskrit terms is important, while 61% of the therapists assert that they actually have such knowledge. 54% think that an Ayurvedic apprenticeship for European Ayurveda therapists should last at least 2 years.

Principal Component Analysis reduced the 12 variables in Table 4 to 3 different factors: (1) factor 1 comprises moksha, dharma, samkhya, vedanta, atman, brahman, and attachment; (2) factor 2 pulls together the concepts of nirvana, enlightenment, attachment, and karma; and (3) factor 3 correlates the concepts of reincarnation, karma, Buddhism, and transmigration of souls (Table 4).

### 3.5. Findings Related to Hypothesis 4

76% of the participants are women; 65% of them are under 50 and above 30 years of age. Among women 65% identify themselves as Christian, among men 43%. Gender differences can also be seen in the answer pattern for the question on whether Ayurveda is spirituality. 81% of women answered “yes,” among men 46% (*P* < 0.001). 91% of the women who consider Ayurveda to be a philosophy also relate it to spirituality (*P* < 0.05). 74% of women think of Ayurveda as a dictum for life, among men only 58% (*P* = 0.148). 50% of men, as compared to 35% of women, deny that Ayurveda is scientific in a modern western sense (*P* = 0.116). 86% of women think that Ayurvedic therapists should be trained by Ayurvedic experts from South Asia, among men 64% (*P* = 0.103). 87% of women believe that Ayurvedic therapists should also have functions related to spirituality, as compared to 65% among men. 64% of the women think that therapists should sympathize with South Asian culture, religion, and philosophy as compared to 50% of men (*P* = 0.390). 73% of the women agree with the statement that a modern western medical education has no negative effects on the religious and spiritual characteristics of therapists, among men 59% (*P* = 0.088). 79% of the women have been involved with rituals (men 66%) and 72% with prayers (men 53%). 85% of the women believe in God, among men 58% (*P* = 0.04). 71% of the women believe in angels, among men 45% (*P* < 0.001). 64% of the male respondents and 44% of the female respondents find Christian religions lacking certain mystic perspectives which, for them, can be found in South Asian religions (*P* = 0.026). 50% of the men think that South Asian religions can offer better solutions to everyday contemporary problems than western religions, among women 43% (*P* = 0.313). When questioned whether South Asian religions play a role for one's partner, 57% of men answered “yes,” while 22% of women answered yes (*P* = 0.002).

## 4. Discussion

The metapostulate of this work was confirmed that individual sociocultural backgrounds, especially religious and spiritual ones, of Ayurvedic therapists and patients influence attitudes and expectations regarding Ayurvedic health care. Statistical relationships between individual religious and spiritual backgrounds and individual decisions to offer or access Ayurvedic services are clearly shown.

A statistically significant larger fraction of women in both groups is noticeable. Both therapists and patients also share an above average education. Results support the thesis that Ayurveda is being used by a predominantly well-educated, urban, and female clientele [76–78]. Differences with respect to income between groups suggest that hybrid forms of Ayurveda in the West are part of a “luxury” medicine; their usage is predominantly reserved for people with higher incomes (see Table 1) [79].

This survey investigates the perception of Ayurveda from a convenience sample of therapists and patients of predominantly western backgrounds. Therefore, it cannot define Ayurveda in any absolute term nor does it attempt to compare contemporary with “classic” Ayurvedic perspectives. Nevertheless the results of this survey point to a conception of Ayurveda as Whole Medical System, which also impacts the implementation of Ayurveda, particularly regarding the patient-doctor relationship [80, 81].

Individual forms of spirituality and religion seem to play a key role in the perception and definition of Ayurveda for patients and therapists. In our population adherers of Ayurveda have a tendency to have a special affinity for Buddhism, Hinduism, and South Asian culture in general. Christian religions (e.g., Protestant or Catholic churches) seem to play a less integral role in the practice and perception of Ayurveda, while “traditional” religious beliefs (e.g., a belief in god, angels, and the devil) can be grouped together with South Asian religious beliefs for a majority of the respondents (notably a belief in god, angels, and the devil can also be included in several South Asian belief systems as more recent texts include such concepts). Spirituality and religious aspects appear to be central in individual conceptions of salutogenesis [82, 83] and within the Ayurvedic therapeutic paradigm [84]. Thereby spirituality, not religion, is the preferred self-categorization within the field of Ayurveda. The results pose the question whether individual references to traditional Christian values might have become weaker due to a loss of confidence in established western religious institutions [85, 86]. These values may thus be substituted or supplemented by the individually composed syncretistic realities of patients and therapists using or offering Ayurveda (e.g., combining god, karma, and nirvana), whose religious and spiritual impulses continue to guide them [87].

While both therapists and patients are engaged with religious and spiritual questions and are open to these issues, therapists seem to deal even more with religious and spiritual matters than their patients. Beyond pure somatic healthcare services, adherents of Ayurveda expect the therapist to also function as a spiritual/psychological caregiver. As a result of training and patient expectations, the Ayurvedic therapist also frequently engages in functions (within an Ayurvedic treatment) that are also characterized by religious and spiritual elements, for example, mantra recitation, performing rituals, meditation, prayers, and so forth [88].

Our data support the hypothesis that elements from South Asian culture, religion, and philosophy seem to play an important role for Ayurvedic patients and therapists. A high level of “authenticity” and “authentic therapy” is expected from the therapists and therapies. It is notable that not only therapists but also patients seem to be quite well versed in South Asian culture, religion, and philosophy. This suggests that the choice for Ayurveda might go hand in hand with a fundamental affinity to South Asian culture and worldview [89].

For Ayurvedic patients and therapists, spirituality, religion, and principles of modern science are not in conflict. For them, Ayurveda contains aspects of spirituality, religion, and science at the same time. While spirituality is seen as a very important aspect, which also influences the daily life of therapists and patients, the medical dimension of Ayurveda is still seen as the most important one and does not exclude the simultaneous use of modern medicine for the majority. The composition of Ayurvedic characteristics that is expected from the majority of those participants could be interpreted as a curiosity for novel things and at the same time as an expression of uncertainty and discontent with prevailing structures. Frustration with modern medicine is less important in the decision to use Ayurveda than, for example, the inclusion of the spiritual dimension. An “enchantment of the world,” a concept often mentioned in CAM contexts, is supposed to help overcome the separation of matter, mind, and soul. Next to scientific knowledge, spirituality stands on equal footing. Religion in a classical sense seems to take a back seat in favour of spirituality. However, this is to a certain degree a tenuous position based on the factor analyses related to the second hypothesis of this work.

In our survey Ayurveda is used—as is CAM in general in the western world—by a well-educated, middle class, and female dominated clientele [90, 91]. Women access Ayurveda more often than men among the surveyed participants, and women appear to be more open to religious and spiritual matters [26]. Almost all characteristics related to religiosity and spiritual attitudes are more prominently represented among women in our data set [92].

Ayurveda patients and therapists seem to be more open to CAM, especially nonwestern CAM methods, but this does not exclude the simultaneous use of modern western medicine for the majority of respondents. Moreover, Ayurveda may be compensating for deficits in the field of psychosocial healthcare logistics [93, 94]. In this conception the Ayurvedic therapist does more than simply treat somatic disorders. Ayurvedic concepts are based on anthropologic/cosmological assumptions which include different levels of human existence in both diagnostic and therapeutic healing approaches. As a result, therapist-patient relationships focused on the individual's unique experience and promoting trust and confidential discussion of spiritual matters in the therapeutic encounter are accommodated and indeed cultivated.

There are several limitations of this work. This study was informed by a small sample size rather than a large scale inquiry; thus various nonspecific effects, for example, the inhomogeneous settings, may have contributed to the answer patterns and thus may have significantly biased the results. Further a statistically significant larger fraction of women in both groups is noticeable (which however also reflects the field). Moreover, the partial reporting of the results as pooled data from patients and therapists may bias the picture depending on potentially different attitudes and knowledge about Ayurveda via patients versus therapists. The fact that 15% of patients are also trained as Ayurvedic therapists is a further limitation and a potential source of bias. Another issue may be that the potential simultaneous use of other CAM methods was not assessed by the questionnaire. Another minor limitation is the fact that some of the used items may have had influencing or directing effects due to their wording or an intentional open phrasing. It is also important to keep in mind while interpreting the results that this not a representative population sample but a sample that was likely to be prone to Ayurveda, which of course is another limitation of this study.

To summarize, key questions regarding the character, essence, complexity, and contextualization of Ayurveda in its original and hybrid forms remain largely unanswered. The following questions yet to be answered seem to be of high exigency. (a) What is Ayurveda in general and can a clear definition of it be given independently of western or Indian contexts? (b) What are the reasons for choosing Ayurveda out of a range of different methods of CAM and is the choice for Ayurveda specific or random? (c) What exactly does “spirituality” mean for therapists and patients in the case of Ayurveda? Overall, normative questions about whether Ayurveda is a science or religion or spirituality seem to be deceptive. It might also be helpful to move away from asking whether and to what extent Ayurveda acts in this context and to instead focus more on why and how it functions in association with science, religion, and spirituality. Let us keep in mind that these concepts are not natural entities. Religion, spirituality, and science are modern western concepts and have a strong potential to export normative and ideological items into what are primarily nonwestern contexts [17, 95–97].

Looking at Ayurveda as a whole medical system including physical, psychological, medical, and spiritual elements, as well as a philosophy and a way of life, may challenge the differentiation, compartmentalization, and rationalization of modern societies [98–100], while leading to a better understanding of Ayurveda as an expression of and complement to “modern western medicine.”

Given the complexity of the topic and the exploratory nature of the survey, larger surveys with fully validated questionnaires, preceding qualitative phases, and refined hypotheses are warranted to support the results of this first pilot survey.

## Figures and Tables

**Table 1:** Sociodemographic data.

Parameters	Therapists	Patients	Total	*P* value
Number of patients (%)	70 (50.0%)	70 (50.0%)	140 (100%)	
Age				0.296
<30	6 (11.6%)	2 (2.9%)	10 (7.2%)	
30–50	42 (60.9%)	50 (71.4%)	92 (66.2%)	
>50	19 (27.5%)	18 (25.7%)	37 (26.6%)	
Gender				0.693
Male	18 (25.7%)	16 (22.9%)	34 (24.3%)	
Female	52 (74.3%)	54 (77.1%)	106 (75.7%)	
Education				0.923
Secondary school	5 (7.1%)	5 (7.1%)	10 (7.1%)	
Junior high school	16 (22.9%)	19 (27.1%)	35 (25.0%)	
High school	13 (18.6%)	11 (15.7%)	24 (17.1%)	
University/college	31 (44.3%)	28 (40.0%)	59 (42.1%)	
Others	5 (7.1%)	7 (10.0%)	12 (8.6%)	
Actual profession				<0.001
Medical doctor	24 (34.8%)	9 (12.9%)	33 (23.7%)	
Alternative practitioner	5 (7.2%)	0 (0%)	5 (3.6%)	
Ayurveda therapist	22 (31.9%)	11 (15.7%)	33 (23.7%)	
Yoga instructor	3 (4.3%)	1 (1.4%)	4 (2.9%)	
Psychologist	1 (1.4%)	0 (0%)	1 (0.7%)	
Medical associated profession	3 (4.3%)	3 (4.3%)	6 (4.3%)	
Others	11 (15.9%)	46 (65.7%)	57 (41.0%)	
Income (*€* per month)				0.233
<1000	17 (25.0%)	7 (10.3%)	24 (17.6%)	
1000–1500	11 (16.2%)	11 (16.2%)	22 (16.2%)	
1500–2000	9 (13.2%)	12 (17.6%)	21 (15.4%)	
2000–2500	7 (10.3%)	6 (8.8%)	13 (9.6%)	
>2500	16 (23.5%)	17 (25.0%)	33 (24.3%)	
Unknown	8 (11.8%)	15 (22.1%)	23 (16.9%)	
Number of children				0.653
0	28 (40.0%)	34 (49.3%)	62 (44.6%)	
1	11 (15.7%)	11 (15.9%)	22 (15.8%)	
≥2	31 (44.3%)	24 (34.7%)	55 (39.6%)	
Location (number of inhabitants)				0.806
<5000	10 (14.3%)	8 (11.6%)	18 (12.9%)	
5000–50 000	18 (25.7%)	17 (24.6%)	35 (25.2%)	
50 000–100 000	11 (15.7%)	8 (11.6%)	19 (13.7%)	
>100 000	29 (41.4%)	35 (50.7%)	64 (46.0%)	
Unknown	2 (2.9%)	1 (1.4%)	3 (2.2%)	

**Table 2:** Characterization of Ayurveda by therapists and patients.

Ayurveda is a…	Therapists	Patients	Total	Total number of valid cases	*P* value
	N (%)		
Health doctrine	67 (100%)	69 (100%)	136 (100%)	136	1
Medical system	66 (97.1%)	57 (91.9%)	123 (94.6%)	130	0.196
Philosophical system	54 (87.1%)	37 (71.2%)	91 (79.8%)	114	0.035
Science	60 (92.3%)	53 (93.0%)	113 (92.6%)	122	0.887
Religious doctrine	16 (30.8%)	18 (36.0%)	34 (33.3%)	102	0.575
Religion	7 (14.0%)	4 (8.5%)	11 (11.3%)	97	0.394
Spirituality	47 (75.8%)	39 (69.6%)	86 (72.9%)	118	0.452
Esoterism	7 (13.7%)	5 (9.8%)	12 (11.8%)	102	0.539
Philosophy of life	39 (73.6%)	34 (66.7%)	73 (70.2%)	104	0.441

**Table 3:** Factor analysis of participants' characterization of Ayurveda.

Variables	Factors			
	1	2	3	4
Ayurveda = spirituality?	**0.8**			
Ayurveda = philosophical system?	**0.8**			
Ayurveda = way of life?	**0.7**			
Ayurveda = religion?		**0.8**		
Ayurveda = religious doctrine?		**0.7**		
Ayurveda = esoterism?		**0.7**		
Ayurveda = medical system?			**0.8**	
Ayurveda = science?			**0.6**	
Ayurveda = philosophy of life?			**0.6**	
Ayurveda = complement to modern medicine?				**0.8**
Ayurveda = closed medical system, which does not require a combination with western medicine?				*−0.7 *
Ayurveda = scientific in a modern western sense?				**0.5**

(Values *x* are omitted, if −0.5 < *x* < 0.5).

**Table 4:** Factor analysis of participants' knowledge of key words of South Asian religion/spirituality.

	Factor		
	1	2	3
Are you familiar with the following term? Moksha	0.9		
Are you familiar with the following term? Dharma	0.9		
Are you familiar with the following term? Samkhya	0.8		
Are you familiar with the following term? Vedanta	0.8		
Are you familiar with the following term? Atman/brahman	0.7		
Are you familiar with the following term? Nirvana		0.9	
Are you familiar with the following term? Enlightenment		0.8	
Are you familiar with the following term? Attachement	0.5	0.7	
Are you familiar with the following term? Attachment			0.9
Are you familiar with the following term? Karma		0.5	0.8
Are you familiar with the following term? Buddhism			0.7
Are you familiar with the following term? Transmigration of the soul			0.5

Extraction method: Main Component Analysis.

Rotation method: Varimax with Kaiser Normalization.

(values *x* are omitted, if −0.5 < *x* < 0.5).
